# Copper Alloy Touch Surfaces in Healthcare Facilities: An Effective Solution to Prevent Bacterial Spreading

**DOI:** 10.3390/ma11122479

**Published:** 2018-12-06

**Authors:** Marius Colin, Flora Klingelschmitt, Emilie Charpentier, Jérôme Josse, Lukshe Kanagaratnam, Christophe De Champs, Sophie C. Gangloff

**Affiliations:** 1 Biomatériaux et Inflammation en Site Osseux, EA 4691, SFR CAP-Santé, UFR de Pharmacie, Université de Reims Champagne Ardenne, 51100 Reims, France; marius.colin@univ-reims.fr (M.C.); flora.klingelschmitt@univ-reims.fr (F.K.); emilie.charpentier@univ-reims.fr (E.C.); jeromejosse18@gmail.com (J.J.); 2 Service de Microbiologie, UFR pharmacie, Université de Reims Champagne-Ardenne, 51100 Reims, France; 3 Unité d’aide méthodologique, CHU de Reims, 51100 Reims, France; lkanagaratnam@chu-reims.fr; 4 Inserm UMR-S 1250 P3Cell, SFR CAP-Santé, Université de Reims Champagne-Ardenne, 51100 Reims, France; cdechamps@chu-reims.fr

**Keywords:** copper, antimicrobial, healthcare-associated infections, long-term care facilities

## Abstract

In the healthcare environment, microorganisms’ cross-transmission between inanimate surfaces and patients or healthcare workers can lead to healthcare-associated infections. A recent interest has grown to create antimicrobial copper touch surfaces, in order to counteract microbial spread in the healthcare environment. For the first time, five French long-term care facilities were at 50% fitted with copper alloys door handles and handrails. Related to the environmental bacterial contamination, 1400 samples were carried out on copper and control surfaces over three years after copper installation. In addition, some copper door handles were taken from the different facilities, and their specific activity against methicillin-resistant *S. aureus* (MRSA) was tested in vitro. In comparison to control surfaces, copper door handles and handrails revealed significantly lower contamination levels. This difference was observed in the five long-term care facilities and it persists through the three years of the study. High and extreme levels of bacterial contamination were less frequent on copper surfaces. Although, the antibacterial activity of copper surfaces against MRSA was lowered after three years of regular use, it was still significant as compared to inert control surfaces. Therefore, copper containing surfaces are promising actors in the non-spreading of environmental bacterial contamination in healthcare facilities.

## 1. Introduction

Among the various problems to deal with in medicalized environment, healthcare-associated infections (HAI) currently represent one of the biggest threats for resident and hospitalized persons all around the world. A prevalence survey estimated the number of HAI in the United States as around 772,000 cases during the year 2011, corresponding to a prevalence of 4% [1]. In 2012, in Europe, the HAI prevalence was around 6%, ranging from 2% to 11% for overall European countries [2]. In the healthcare environment, microorganisms can easily spread from resident to resident [3] and from resident to staff through different pathways. Direct contact between people is the most obvious factor of microbial cross-contamination. The hands of a healthcare worker can have a high risk of being contaminated following a direct skin contact with infected patient [4,5,6], and hand hygiene is the first strategy for preventing healthcare-associated infections [7]. However, pathogens can also spread through contact between people and touch surfaces. Indeed, by multiple surface-skin transfer, a contamination on a primary surface can somehow very quickly spread across an entire service or care unit [8]. This observation highlights the role of the surfaces in the dissemination of microorganisms and it suggests that surfaces may represent sources of microbial contamination for the users [8,9,10]. Furthermore, most of the microorganisms, and more specifically bacteria, can persist on these surfaces for an extremely long time [11,12]. To hamper this situation, the main action against bacteria persistence remains the regular cleaning and disinfection of touch surfaces [12]. However, as observed, bacteria can rapidly recolonize the surfaces after disinfection. For example, 24 h after decontamination with hydrogen peroxide vapor, methicillin-resistant *Staphylococcus aureus* (MRSA) have been detected on surfaces in an intensive care unit [13].

Nowadays, many approaches other than cleaning and disinfection are proposed to reduce the ability of microorganisms to adhere, survive, and grow on surfaces. Among them, the addition of active compounds at the surface of different materials and equipment takes a large part. One other approach is to work with inorganic materials having inherent antimicrobial properties, such as silver [14], TiO_2_ photocatalysts [15,16,17], and copper. The capacity of copper surface to kill a wide range of bacterial, viral, and fungal species was highlighted through in vitro tests, demonstrating the antimicrobial activity of copper ions [18], oxides [19], and solid copper surfaces [20,21,22,23,24,25,26,27,28] under specific conditions. The key of copper antimicrobial activity is ions releasing from the surface [29]. On dry metallic copper surfaces, copper ions are released from the surface due to environmental humidity. Nevertheless, the contact between copper surface and bacterial cell wall components seems to be sufficient to trigger a release of copper ions from the surface [30,31]. Copper ions will then induce damages to bacterial cell wall and membranes, production of reactive oxygen species, and degradation of DNA, resulting in rapid cell death [29].

Nevertheless, the persistence of the antibacterial activity of metallic copper surfaces is still in debate. In this study, the ability of in situ copper alloys door handles and handrails to reduce the bacterial burden in five French long-term care facilities was investigated over a three years period of time. In addition, in vitro tests with MRSA were done on copper door handles that were collected in these long-term care facilities at different moments.

## 2. Materials and Methods 

### 2.1. In Situ Approach: Environmental Contaminations of Frequently Touch Surfaces

#### 2.1.1. Long-Term Care Facilities Environment and Study Design

The study was conducted in five long-term care facilities located in the Marne territory, France, named A–E afterwards in the text: (A) E.H.P.A.D. Villa des Rèmes (Korian, Reims), (B) E.H.P.A.D. Sarrail (Centre Communal d’Action Sociale, Châlons-en-Champagne), (C) M.A.R.P.A. Les Charmilles (Association de Gestion de la M.A.R.P.A., Courtisols), (D) E.H.P.A.D Saint-Joseph (Maison Saint-Joseph, Châlons-en-Champagne), and (E) E.H.P.A.D Wilson (Centre Hospitalier Universitaire, Reims). Each facility employs its own staff and uses its own protocol for disinfection and cleaning. Those protocols have not been changed throughout the study. In each facility, half of the resident’s room and corridors were fitted out with Steriall^®^ copper alloys (Lebronze alloys, Suippes, France). Briefly, half of the original door handles of residents’ room and original corridors’ handrails were replaced by copper ones, the other half remained as before and was used as control surfaces. Two different copper alloys were used, one for the rooms’ door handles (around 90% of copper) and one for the handrails (around 70% of copper) in every corridor adjoining copper fitted rooms. The sampling series were conducted over an 18 months period and executed with at least one week of delay between them. The number of rooms involved, surface cleaning protocol and frequency, date of the copper alloys set-up, and sequences of sampling are summarized in Table 1.

For each series, ten copper as well as ten non-copper fitted patient rooms were randomly selected and excluded from the next series of the same sequence (except in facility C were the total number of rooms was below 30). For each selected room, the samplings were performed on the external door handle (corridor side) and on the closest handrail in the corridor, at a distance of 10 centimeters from the edge of the handrail. 

#### 2.1.2. Sampling Protocol

Samplings were performed on Monday or Tuesday between 7:30 a.m. and 9 a.m., during staff operating time and before surfaces daily cleaning. Each door handle or handrail was sampled using a 10 cm^2^ (2 cm × 5 cm) sterile silicon template and a sterile swab (Copan, Murrieta, CA, USA). The swab was moistened in sterile peptone water (Sigma-Aldrich, Dutscher, Brumath, France) and was applied five times longitudinally and 15 times transversally. Then after, each swab was immediately transferred in a 15 mL tube (Falcon, Dutscher, Brumath, France) containing 1 mL of sterile peptone water. Samples were stored at 4 °C during transport to the laboratory and were then inoculated on agar plates’ surface within the 6 h following sampling.

#### 2.1.3. Bacteria Recovery and Culture

To release the collected bacteria from the swab to the resuspension medium, samples were placed in ultrasonic bath at 35 kHz for two minutes, and then vortexed for 30 s. Hundred µL of each sample were then inoculated onto tryptic soy agar plates (TSA, Biokar Diagnostics, Allonne, France). Plates were incubated aerobically at 37 °C. The number of bacterial colony-forming units (CFU) on each plate was determined after 24 h and confirmed after 48 h and 72 h. The lower detection limit was one CFU per cm^2^.

### 2.2. In Vitro Approach: Anti MRSA Standardized Activity of “in Use” Copper Door Handles

#### 2.2.1. Door Handle Collection

The antimicrobial activity of the door handles on MRSA was studied in vitro on different sets of door handles (Lebronze alloys, Suippes, France). Unused door handles came directly from the factory. Used door handles were collected from the five facilities approximately one year (beginning of the in situ study) or three years (end of the in situ study) after their set-up in the long-term care facilities. In order to preserve the characteristics of the in-use copper door handles, they were carefully removed from the doors of the residents’ room.

#### 2.2.2. Preparation of the Handles

To avoid any changes in the characteristics of the handles, no drastic physical or chemical treatments were performed on their surface before the in vitro tests. Three hours before the antibacterial activity assay, a sterile swab was moistened with 50 µL of peptone water and was applied gently on all the surface of the handle to remove non-adherent particles if any. Glass slides (Ghäasel, Dutscher, Brumath, France) and stainless steel door handles (Trenois Decamps, Wasquehal, France) were used as control surfaces.

#### 2.2.3. Methicillin-Resistant *Staphylococcus aureus* Strain Preparation

The MRSA strain CIP 103811 was maintained for long-term conservation at −80 °C in glycerol in cryotubes and thawed just before use. Two hundred µl were resuspended in 25 mL of tryptic soy broth (TSB, Sigma-Aldrich, Dutscher, Brumath, France) and incubated at 37 °C over night. Ten µL of the culture were then stripped on TSA to create a reference petri dish. The strain purity was checked by Gram staining on colonies, and the resistance to methicillin was confirmed by the disc diffusion method according to the European Committee on Antimicrobial Susceptibility Testing guidelines [32]. For each assay, a single colony was resuspended in 25 mL of TSB and incubated at 37 °C over night. Subsequently, 2 mL of the culture were added to 50 mL of TSB, and incubated at 37 °C, stirring at 250 rpm, during four hours. After three washes with 10 mL peptone water and centrifugation (3000 g, 5 min), the bacterial pellet was resuspended in peptone water to reach a concentration of 10^10^ CFU/mL.

#### 2.2.4. Bacterial Viability on Door Handles

Under sterile conditions, three independent droplets of 10 µL (around 10^8^ CFU) were inoculated on the tested surface and then incubated for two hours at room temperature. Bacteria of each inoculum were then carefully harvested using a sterile swab moistened with 50 µL of peptone water. The swab was firmly applied on area of the inoculum and rotated. The swab was then placed in a 50 mL sterile tube containing 7.5 mL of peptone water. Tubes were placed in ultrasonic bath (35 kHz) during 2 min and then briefly vortexed for 30 s. Serial dilutions of each tube suspension were performed in peptone water and 100 µL of each dilution was exponentially seeded (easySpiral Pro, Interscience, Saint-Nom-la-Bretèche, France) on TSA. Plates were incubated at 37 °C for 24 h. Colonies were counted with the Interscience Scan 1200 to determine the number of viable MRSA remaining in each inoculum. For each test, these numbers were streamlined to the mean number of viable MRSA remaining on the glass slide.

### 2.3. Statistical Analysis

The non-parametric Mann-Whitney test was used to compare the copper and control surfaces, in both in vitro and in situ analysis, using the StatXact software (version 7.0.0, Cytel studio, Cambridge, MA, USA). Kruskal-Wallis was used to compare contamination between the five long-term care facilities, also using the StatXact software. For every test, a *p*-value < 0.05 was considered as statistically significant (two-sided). 

## 3. Results

### 3.1. Global Dispersion of the Bacterial Contamination

**Figure 1:**
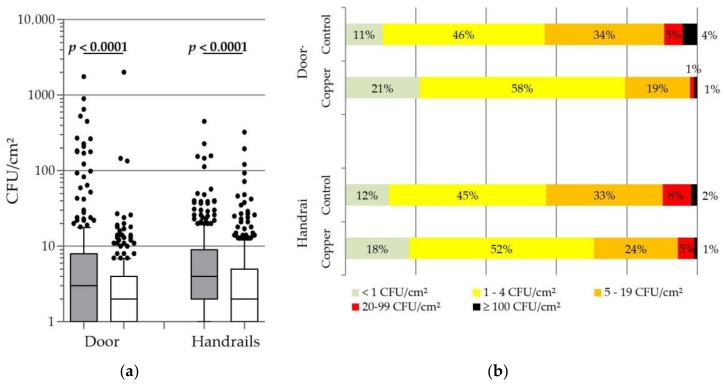
Distribution of the environmental bacterial burden found on touch surfaces. Overall compilation for the five long term care facilities. (**a**) Box plot representation of all the values. Grey boxes, control; White boxes, copper. Horizontal line in box, median; Boxes extremities, 1–4 quartiles; Whiskers, 10–90 percentiles; dots, 10% highest values; Significant *p*-value of Mann-Whitney U test are noted in bold. (**b**) Frequency distribution of the bacterial burden concentration (%). Door handles: *n* = 341. Handrails: *n* = 347.

During the sampling sequences in the five long-term care facilities, a total of 688 copper surfaces and 688 control surfaces were sampled, with 682 samples being obtained from door handles of resident’s room and 694 obtained from handrails in corridors. As seen in Figure 1a, the profiles obtained for the door handles and the handrails are similar, the bacterial burden being significantly lower on copper surfaces than on control surfaces. Copper door handle showed an average of 59% reduction (median difference, −1.0 CFU/cm^2^), while copper handrails showed an average of 33% reduction (median difference, −2.0 CFU/cm^2^). Looking more specifically at the frequencies of low and high levels of contaminations (Figure 1b), we can see that the copper door handles and handrails are much less frequently contaminated than the controls. Looking at very low contaminations, 21% of the copper door handles showed less than 1 CFU/cm^2^ on their surface, as compared to only 11% for the control door handles. Looking at the high contaminations, superior to 20 CFU/cm^2^, only 2% for the copper alloys handles, while more than 9% of the control door handles were concerned. Furthermore, extreme contaminations, defined as sample presenting ≥100 CFU/cm^2^, have been observed but occurred significantly less frequently on copper surfaces [three copper door handles (1%) vs. 14 control door handles (4%) and three copper handrails (1%) vs. six control handrails (2%)].

### 3.2. Bacterial Contamination by Long-Term Care Facility

**Figure 2:**
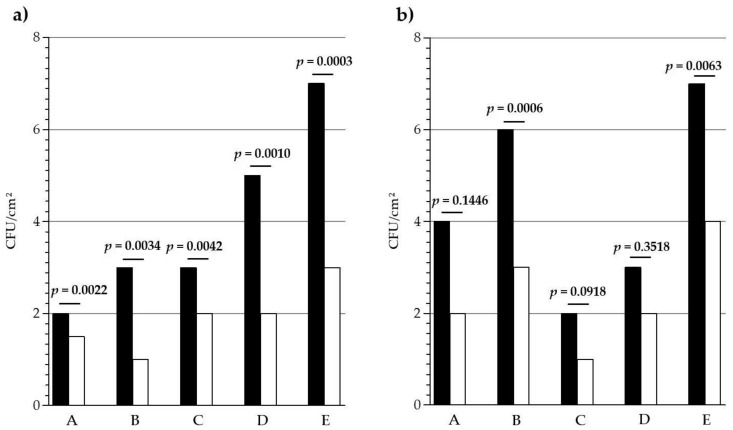
Bacterial burden recovered from (**a**) door handles and (**b**) handrails in each long-term care facility (A to E). Black bars, control; White bars, copper. 65 ≤ *n* ≤ 70. Median values.

To get a more precise view, the contamination distribution in each long-term care facilities was analyzed independently. As seen in Figure 2, the surface contamination was different from one establishment to another, with significant differences between the five long-term care facilities (*p* < 0.0001). Nevertheless, in each facility and for both door handle and handrail, the median bacterial burden was lower on copper surface than on the control. Concerning door handles (Figure 2a), the differences between copper and control median burdens were significant in every establishment, and ranged from 0.5 CFU/cm^2^ (A) to 4.0 CFU/cm^2^ (E). For handrails, the median differences ranged from 1.0 CFU/cm^2^ (C and D) to 3.0 CFU/cm^2^ (B and E) and they were statistically significant only for B and E. 

### 3.3. In Situ Persistence of the Copper Antibacterial Activity

**Figure 3:**
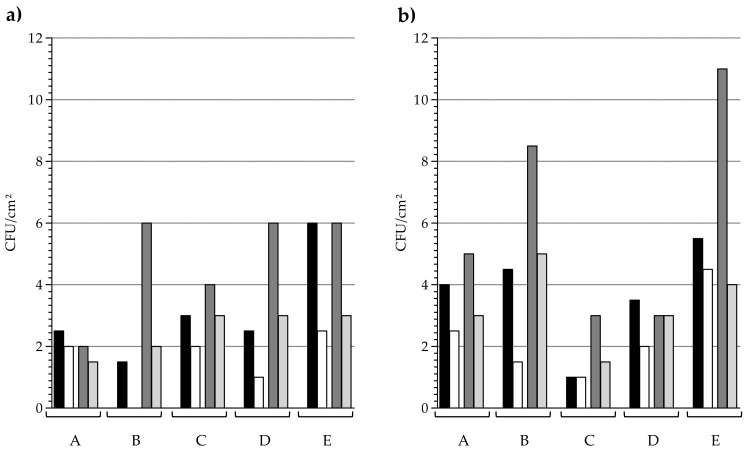
Sequence analysis: Bacterial burden recovered from (**a**) door handles and (**b**) handrails in each long-term care facility (A to E). Black bars, control sequence 1; White bars, copper sequence 1; Dark grey bars, control sequence 3; Light grey bars, copper sequence 3. 26 ≤ *n* ≤ 30. Median values.

As different series of sampling have been realised over the three years in each long-term care facilities, the persistence over time of the mean antibacterial activity was analysed (Figure 3). Looking at the door handle (Figure 3a), a stronger disparity could be observed in contaminations sampled from control elements than from copper elements. Comparison between copper and control surfaces revealed that, in each long-term care facilities and for both sequences, the median bacterial burden was lower on copper door handles. Looking at the median bacterial burden on handrail (Figure 3b), it was once again systematically lower on copper, except for one series for C and D, where the median on copper and control surfaces were equal.

### 3.4. In Vitro Evaluation of Door Handles Activity against MRSA

**Figure 4:**
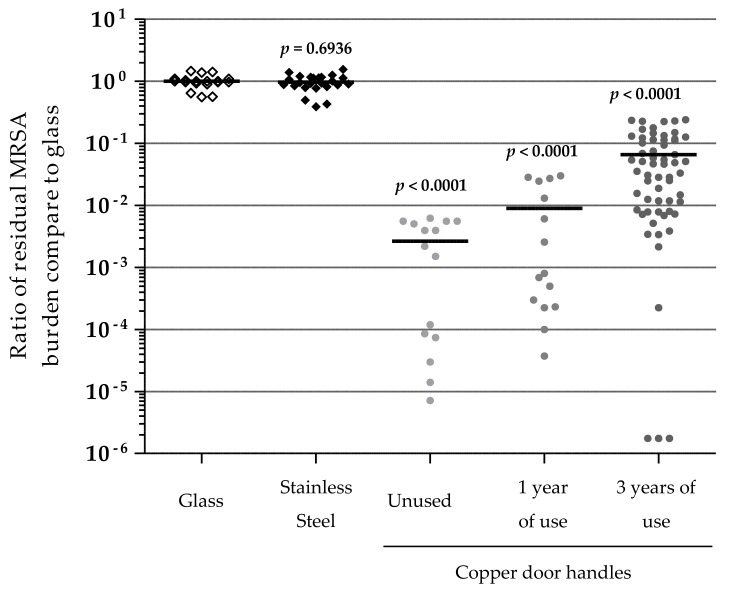
Residual methicillin-resistant *Staphylococcus aureus* (MRSA) burdens after 2 h of contact with the surfaces. The tested surfaces were stainless steel, and copper door handles after different times of use in long-term care facilities (unused, 1 year and 3 years after set-up). All the values are the ratio of the number of colony-forming units (CFU) observed on tested surfaces over the mean of CFU observed on glass as reference. Black lines represent the mean values of raw data. *p*-values of the comparison of each type of surface with glass.

One and three years after copper alloys set-up, several copper door handles, randomly selected, were removed from the five long-term care facilities and transferred to the laboratory to evaluate their efficiency against a MRSA strain. In vitro tests were also performed on unused copper door handles, stainless steel door handles, and glass slides, to compare the residual bacterial burden after two hours of contact (Figure 4). No significant difference was observed in the distribution of the residual burden that was recovered from glass slides or stainless steel door handles. Even so, the MRSA burden was significantly reduced as compared to control; there was more heterogeneity in the residual burden distribution on the copper handles. Unused copper door handles showed an average 3.2 (±0.6) log reduction compared to glass, while one year used copper handles showed a 2.7 (±0.7) log reduction average, and the three years used copper handles a 1.7 (±1.1) log reduction average. The door handles that were used for three years presented the higher heterogeneity with efficiency ranging from 5.7 log reduction to 0.7 log reduction. 

## 4. Discussion

The spread of infections in healthcare facilities is an accurate problem, notably through inanimate surfaces contamination [8,33]. A simple gesture such as “holding a door handle” can participate to a wide and fast dispersion of pathogens and favor the cross-contamination between inanimate surfaces and patients. Indeed, inanimate surfaces play an important role as a microbial beholder, despite hand-washing, which currently remains the primordial step against pathogens dissemination. To fight against microbial dissemination, new disinfection methods are being developed. Nevertheless, bacteria can still rapidly recolonize touch surfaces, especially multidrug resistant bacteria that are frequently found in healthcare facilities [13]. Given this context, the use of copper surfaces brings a new perspective for constant and inherent disinfection. In this study, we investigated the antimicrobial properties of door handles and handrails containing copper that have been used on a daily base in five long-term care facilities.

Independently of the type, size, or localization of the establishments (city, countryside), the median contaminations levels observed were always lower on copper surfaces than on the control surfaces (Figure 2). The estimate average bacterial burden reduction on copper was 59% for door handles and 33% for handrails. When compared to similar in situ investigations [34,35,36,37,38,39,40,41,42,43], these reduction levels may seem lower than those that were observed in several other studies [34,35,36,37,41,42] reaching, for some types of furniture, 90% of reduction [41], but obviously, several different experimental points between these studies and ours can potentially explain these differences. 

First, those studies mainly focused on hospital wards, while our study focused on five long-term care facilities, where different types of peoples are moving and meeting (patients-residents/staff/visitors). Different human populations can convey different microbiomes and bacterial burdens, directly impacting bacterial contamination on touch surfaces.

Second, the sampling protocols are quite different. The difference in sampling protocols may induce performance variations in the bacterial recovery. To collect bacteria from touch surfaces, we used a moistened cotton swab. Among others techniques, such as contact agar plates or wipes [35,37,40,41,42], swabbing is preferred to perform samplings on small area and non-flat surfaces [34,37,38,43], like the door handles and handrails of this study. Due to the size and form of the copper door handles, the area of 10 cm^2^ was used here as the standard area to sample, which is a smaller surface than in several other studies where sampled areas were frequently up to 100 cm^2^ or more [35,36,37,40,41,42]. Also, peptone water was used as resuspension medium for bacteria, while other studies used media like saline water (0.9%) [34,35,36,37,38] or neutralizing buffers [35,36,37,40,42]. All of these experimental factors combined can lead to differences in the results.

Third and maybe the most important, copper alloys compositions are probably very different from one study to another. Even if the percentage of copper is known, minor elements can have a huge importance in the antimicrobial efficiency of the alloy [23,24].

In our study, the cleaning protocols remained unchanged after the copper surfaces set-up in four of the five long-term care facilities and are different when compared to those that were used in other studies [34,35,36,37,38,39,40,42,43]. Indeed, the regular use of classic disinfectants or cleaning solutions can somehow modify the copper surface and lead to a loss of the surface antibacterial activity. Such antagonist effects between cleaning solutions and copper surfaces have already been pointed out [39,44]. Mikolay et al. [39] observed low bacterial burden reductions by copper surface, ranging from 0% to 40%. They suggested that these values were due to the daily cleaning solution used in the hospital, proposing that the glucoprotamin, a bactericidal compound that is present in the solution, may form a thin layer on copper surface, thus protecting environmental bacteria from direct contact with copper. These results indicate that the use of an appropriate disinfectant or cleaning solution for copper surfaces and the application of a specific upkeep protocol could lead to an even better and stable copper antimicrobial activity.

Looking individually at the five establishments, singularities in the bacterial burden levels have been observed. Highest global contaminations were found in long-term care facilities E, on both control (median of door handles, 7 CFU/cm^2^; median of handrails, 7 CFU/cm^2^) and copper (median on door handles, 3 CFU/cm^2^; median on handrails, 4 CFU/cm^2^). Interestingly, it is, by far, the largest long-term care facility of the study, with a capacity of 347 residents and a number of 193 employees. Potentially, a higher number of people in an establishment may induce a more intensive use of surfaces, and then results in higher levels of surface contaminations, but also on a faster lowering of the antibacterial activity of copper by oxidation or dirt accumulation. 

The values of bacterial burden were very variable between the establishments and inside each establishment, ranging from <1 CFU/cm^2^ to >2000 CFU/cm^2^. High-level contaminations, depending on numerous parameters (humidity, temperature, time delay between contamination, and sampling) stochastically occurred on touch surfaces in long-term care facilities during the study. Our results showed that copper surfaces are less subject to these extreme contaminations, this frequency being reduced by 50% on copper handrail and by 79% on door handles. The average reductions of bacterial burden, as well as the 4,6 times decrease of high contaminations (>20 CFU/cm^2^) on copper door handles as compared to control handles, (Figure 1) suggest that door handles display a stronger antibacterial activity than handrails. These differences in activity between the door handles and handrails may depend in many factors, including the cleaning protocols that are different for door handle and handrail, the way the two types of items are used by the persons and most probably the difference in the percentage of copper, door handles having a higher copper percentage (90% vs. 70%) and being more active against bacteria than handrails.

No correlation was highlighted between contaminations levels and external temperature when sampling was carried out (data not shown). Nevertheless, it cannot be excluded that the period of the year has a slight effect on the global bacterial populations (temperature, humidity, wintry and summer infections, percentage of sick residents). In addition, statistical correlation indicates that the medians of bacterial burden on copper surfaces correlates with the medians that were observed on control, demonstrating that the global bacterial burden levels on touch surfaces are influenced by forces depending on the facility and the period of time. Taking all into account, the overall bacterial burden levels were lower on copper surfaces, and this was observed in the five establishments, one year and three years after copper set-up, for both door handle and handrails (Figure 3). Thereby, regardless of the healthcare facilities, copper surfaces maintained their antibacterial activity. 

To see if this antibacterial activity was still relevant against MRSA, in vitro contaminations were assayed (Figure 4). MRSA is a pathogen that is frequently involved in healthcare-associated infections [2,45] and is regularly found on healthcare touch surfaces [46], where it can potentially persist under environmental conditions for months [12]. Here, we highlighted that, after three years of normal use in healthcare facilities, the copper handles maintain their activity against this pathogen, with a reduction superior to 90% for most of the tested copper door handles, as compared to the residual burden on glass and stainless steel door handles. The efficiency of the copper surfaces, however, seems to slowly decrease with time. While new copper door handles allowed an average 3.2 logs reduction of number of MRSA within two hours, the reduction dropped to 2.7 logs after one year and 1.7 logs after three years of use. Still, copper surfaces remain an interesting long-term solution to fight against touch surface contaminations, especially if installed in the whole facility. Also, as mentioned before, the cleaning protocols remained identical than before the set-up of copper surfaces and other studies have already suggested that cleaning solutions may have an antagonist effect on copper antimicrobial activity [39,44]. Yet, even if using these inadequate solutions for more than three years, copper surfaces still demonstrate a significant impact against MRSA.

## 5. Conclusions

This study shows for the first time in long-term care facilities the antibacterial activity of Steriall^®^ copper alloys over more than three year of use, as well as the persistence of their activity against MRSA. Although some ameliorations can be proposed in term of cleaning solution or protocols to keep the activity at its highest level, these copper surface already play an effective role in the thwarting of the bacterial spread, even in non-optimal use. Working on the aging of the copper surface could bring new insight to further increase the antimicrobial activity of the copper alloys and to develop copper surfaces that completely sustain this activity over years of use.

## Figures and Tables

**Table 1:** Long-term care facilities: Key parameters for the setting of the study.

Long-Term Care Facilities	A	B	C	D	E
**Total Number of rooms**	82	117	24	56	347
**Copper outfitted rooms**	43	54	12	30	158
**Number of employees (Full-time equivalent)**	55	71	6	33	193
**Control handles**	PVC	PVC	Stainless steel	Aluminium or PVC	PVC
**Control handrails**	Wood	PVC	PVC	Aluminium or Wood	Wood
**Surface cleaning solution used**	Diesin HG ^a^	APESIN Clean Bacto ^b^	D10.1 ^c^	Helispray A ^d^ or water on copper surfaces	Aniosurf ^e^
**Surface disinfection frequency**	1/day	1/day	1/day	1/day	1/day
**Copper installation date**	07/2014	07/2014	10/2014	06/2014	06/2014
**Seq 1 dates**	06 June 2016 13 June 2016 20 June 2016	07 June 2016 14 June2016 21 June 2016	28 June 2016 04 July 2016 18 July 2016	27 March 2017 03 March 2017 10 April 2017	03 October 2016 10 October 2016 17 October 2016
**Seq 2 date**	06 February 2017	13 February 2017	13 February 2017	06 June 2017	16 June 2017
**Seq 3 dates**	11 September 2017 18 September 2017 25 September 2017	26 September 2017 03 October 2017 10 October 2017	02 October 2017 09 October 2017 14 November 2017	16 October 2017 06 November 2017 13 November 2017	20 November 2017 27 November 2017 04 December 2017

The providers of the cleaning solutions are: ^a^ Ecolab (Issy Les Moulineaux, France); ^b^ Werner & Mertz (Les Ulis, France); ^c^ Diversey (Fontenay-sous-bois, France); ^d^ Hydrachim (Le Pertre, France); ^e^ Laboratoires Anios (Lille, France).
